# The current status and trend of the functional magnetic resonance combined with stimulation in animals

**DOI:** 10.3389/fnins.2022.963175

**Published:** 2022-09-23

**Authors:** Jiayang Huang, Yusi Zhang, Qi Zhang, Linxuan Wei, Xiwen Zhang, Caiping Jin, Junchao Yang, Zuanfang Li, Shengxiang Liang

**Affiliations:** ^1^National-Local Joint Engineering Research Center of Rehabilitation Medicine Technology, Fujian University of Traditional Chinese Medicine, Fuzhou, China; ^2^Rehabilitation Industry Institute, Fujian University of Traditional Chinese Medicine, Fuzhou, China; ^3^College of Rehabilitation Medicine, Fujian University of Traditional Chinese Medicine, Fuzhou, China; ^4^Basic Medicine College, Hebei University of Chinese Medicine, Shijiazhuang, China; ^5^Innovation and Transformation Center, Fujian University of Traditional Chinese Medicine, Fuzhou, China; ^6^Traditional Chinese Medicine Rehabilitation Research Center of State Administration of Traditional Chinese Medicine, Fujian University of Traditional Chinese Medicine, Fuzhou, China

**Keywords:** functional magnetic resonance imaging, stimulation, animal, brain, activation

## Abstract

As a non-radiative, non-invasive imaging technique, functional magnetic resonance imaging (fMRI) has excellent effects on studying the activation of blood oxygen levels and functional connectivity of the brain in human and animal models. Compared with resting-state fMRI, fMRI combined with stimulation could be used to assess the activation of specific brain regions and the connectivity of specific pathways and achieve better signal capture with a clear purpose and more significant results. Various fMRI methods and specific stimulation paradigms have been proposed to investigate brain activation in a specific state, such as electrical, mechanical, visual, olfactory, and direct brain stimulation. In this review, the studies on animal brain activation using fMRI combined with different stimulation methods were retrieved. The instruments, experimental parameters, anesthesia, and animal models in different stimulation conditions were summarized. The findings would provide a reference for studies on estimating specific brain activation using fMRI combined with stimulation.

## Introduction

Since the 1930s, magnetic resonance imaging (MRI) has been used as an effective tool for neuroscience research *in vivo*. Seiji Ogawa discovered the MR contrast mechanism of functional magnetic resonance imaging (fMRI) in 1990 (Ogawa et al., 1990). It relies on blood oxygen level-dependent (BOLD) changes in brain tissue. With the development of magnetic resonance technology, this oxygenation contrast becomes more obvious in high spatial resolution magnetic resonance imaging, which makes fMRI a non-radiative and non-invasive technique for studying neural activity changes (Liang S. et al., 2017; Zhang et al., 2021). In the initial human sensory stimulation studies, scientists realized that this was a reasonable way to map brain function (Ogawa et al., 1992).

fMRI can reflect the fluctuation of blood flow in the brain, which could map the patterns of brain activity in the resting state, the directional activation of specific brain regions, or the pathways caused by external stimulation (Sanganahalli et al., 2009). Humans and animals are connected to their environment (Arora, 2021). They receive information from the outside world all the time and send it to the brain for analysis (Ferezou and Deneux, 2017), which is the basis for human beings to perceive the world. Resting-state fMRI studies are meaningful, but evoked-state fMRI is preferred in dynamic interactions because it can reflect BOLD changes in the brain from multiple dimensions (Han et al., 2019). As early as 1995, Yang et al. (1996) performed vibration stimulation on the rats' whiskers to reflect the changes of external stimulation in the brain. A series of studies focusing on different stimulation modes has gradually emerged with the development of science and technology. Early studies have used electrical (Scanley et al., 1997), visual, olfactory, and auditory stimulation (Jezzard et al., 1997). Later, optogenetic, deep brain stimulation, electrical microstimulation, and multiple stimulations emerged (Kamada et al., 1999; Ferris et al., 2001). A variety of stimulation paradigms have been devised to accurately reflect the different brain regions and pathways through which animals communicate with the outside world, and the application of the stimulation provides a dependent variable for fMRI studies (Sanganahalli et al., 2009). Since stimulation intensity, location, electrode selection, and stimulation method may all affect imaging quality and stability (Spenger et al., 2000; Luo et al., 2009; Zhao et al., 2020), it is particularly important to design a suitable, reliable, and repeatable paradigm to meet the specific experiment objective (Chen et al., 2020).

In this review, we aimed to describe the current status and trends of animal fMRI combined with stimulation. Both the stimulation paradigms and the corresponding changes in fMRI activation during stimulation were explored *via* collecting and analyzing the activation states, the facility information, and the relevant information of stimulations in the previous animal fMRI studies. The findings would provide a basis for the choice of the stimulation paradigm and the parameter settings of fMRI to explore brain activation in animal studies.

## Methods

We searched for the relevant articles in the PubMed database on September 25, 2021, using the following searching strategy. “Search (functional magnetic resonance imaging [Title/Abstract]) or (fMRI [Title/Abstract]) or (functional MRI [Title/Abstract]) and (stimu^*^ [Title/Abstract]) Sort by: Best Match Filters: Abstract; Filters: Other Animals, from 1992–2021; Other Animals.” We retrieved a total of 1,556 research articles. After screening the titles and abstracts, we excluded studies that did not use stimulation in the fMRI scanning process and received a total of 667 articles in the end. We extracted all kinds of parameters related to the stimulation methods, species, and anesthesia methods.

## Results and discussion

### Year distribution

**Figure 1:**
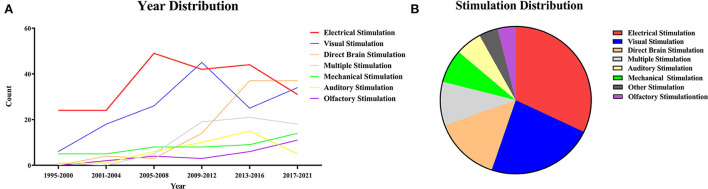
**(A)** Year distribution of stimuli. The years of the collected articles were sorted out according to the types of stimuli and then plotted into a line graph of year distribution. The line graph can intuitively reflect the hot spots and trends of the research on stimulation combined with functional magnetic resonance. **(B)** Stimulation distribution. All stimulation is divided according to the method of the stimulus, including electrical stimulation, visual stimulation, direct brain stimulation, multiple stimulation, mechanical stimulation, olfactory stimulation, auditory stimulation, and others. Among them, electrical stimulation (n = 214, 32.08%), visual stimulation (n = 154, 23.09%) and direct brain stimulation (n = 95, 14.24%) are the main methods.

The imaging quality of fMRI is related to the strength of the magnetic field and the scan sequence. As early as 1995, relevant research studies had already been performed, and they were reproducible in and among animals within the experimental error range (Yang et al., 1996). The specific time distribution of the studies is shown by the broken line graph (Figure 1A). “Most studies were” means that most of the early experiments were centered around electrical, visual, and mechanical stimulation. And as you can see from the year distribution (Figure 1A) these three types of stimuli have been studied earlier. With the development of optogenetics, deep brain stimulation (DBS), and electrical microstimulation (EM), there has been an increasing number of related fMRI studies because of their compatibility. Besides, the number of multistimulation experimental studies was increasing. At the same time, two of the most frequently used methods of experimental stimulation in the past—electrical and visual stimulation—showed a downward trend. Among other stimulations, the number of studies using direct brain stimulation and combined multistimulation has increased, while the number of studies using olfactory, auditory, mechanical, and other types of stimulation showed no obvious change. It indicated that multistimulation and direct brain stimulation are likely to be used more often in future studies.

### Methods of animal anesthetization for scanning

**Figure 2:**
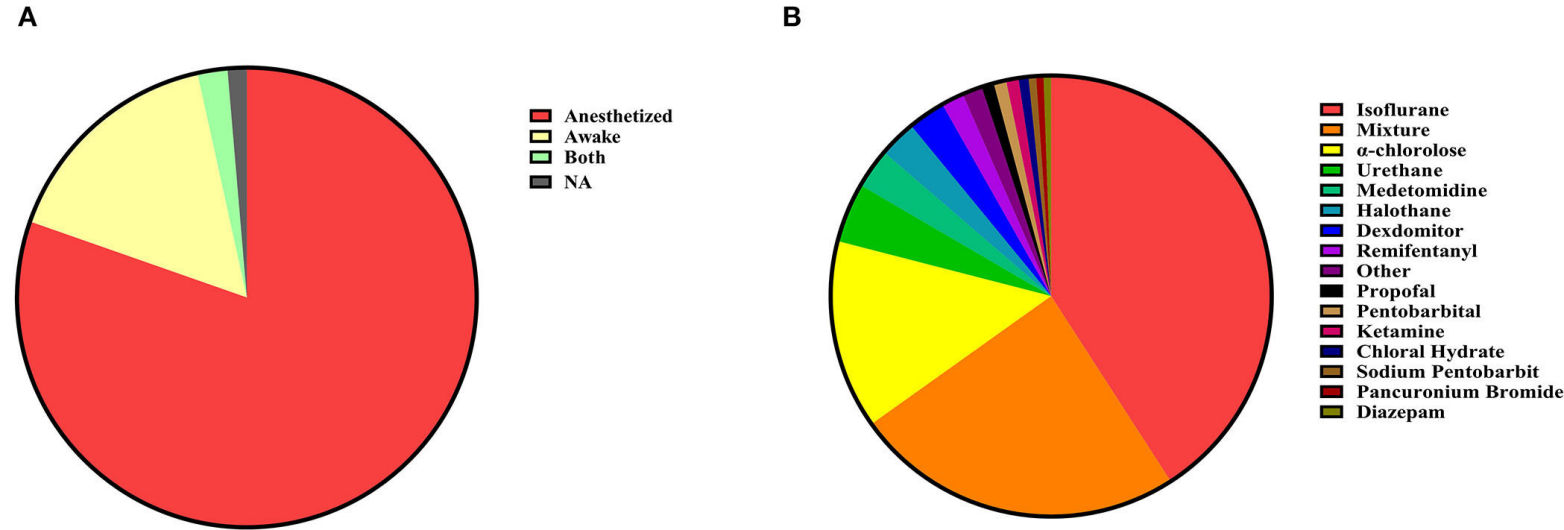
**(A)** Animal preparation. Animal fMRI relies mainly on anesthesia to help restrain animals; most studies are still conducted under anesthesia (n = 536, 80.36%). Compared with numerous articles under anesthesia, the awake experiment is more valuable (n = 108, 16.19%). A very few contained both animal states (n = 9, 1.35%) and unstated articles (NA, n = 14, 2.10%). **(B)** Anesthetics distribution. Isoflurane is the most commonly used anesthetic in experiments related to anesthesia, and the combination of anesthetics is also a popular choice.

During MRI scanning, the state of the animal can be classified as awake or anesthetized (Figure 2A). Awake imaging does not need an anesthetic during the scan, which usually achieves stable imaging through early adaptive training and restriction devices in different animals (Ferris, 2022). Under certain circumstances, a faster scan sequence (fast low-angle shot, FLASH) may be used to obtain a sharper image (Tsurugizawa et al., 2009b, 2010). While anesthesia imaging needs to maintain stable imaging of animals with an anesthetic, inhalation of isoflurane is the most commonly used anesthetic method in anesthesia experiments. Alpha-chloralose is also a more common choice. The choice of anesthetics is not limited to a single type, but mixed anesthesia (multiple mixed uses) is also often used. Ketamine is often used in a mixed way, and there are fewer ways to use ketamine alone (Figure 2B).

Anesthetizing the experimental animals is more time-saving, labor-saving, and convenient for intervention than dealing with them in an awake state. Therefore, anesthetized animal models are preferred in research studies. For example, the neurovascular coupling has been studied extensively in anesthetized animal models. However, it showed severe disruption of brain metabolism, neural activity, and cardiovascular function. When the rodent models are anesthetized, fMRI scanning is a common method, but the physiological parameters of animals might change in the anesthetized state, which would affect fMRI results. With the emergence of different scanning schemes, designs, and modifications of scanning beds, it is now possible to study neurovascular coupling and brain circuit function in animals when they are awake and behaving normally. As long as these factors are not objectively quantified, the scientific validity of fMRI application in rodents will be compromised (Steiner et al., 2021). The imaging process of fMRI in the awake state is also worth further study, as it is closer to a normal physiological state. Some studies on the wakefulness state have been carried out in different animals, which showed repeatable stability (Hirano et al., 2018).

### Sensory stimulation

The distribution maps of different stimulation types were plotted through data statistics (Figure 1B). fMRI studies with stimulation can be divided into electrical, mechanical, multiple, visual, olfactory, direct brain, and other kinds of stimulation based on the type and location of stimulation. The different stimulation methods will be elaborated on separately in the following sections.

The studies were categorized according to the method of the stimulation, including electrical (n = 214, 32.08%), visual (n = 154, 23.09%), direct brain (n = 95, 14.24%), multiple (n = 64, 9.60%), mechanical (n = 49, 7.35%), auditory (n = 38, 5.70%), olfactory stimulation (n = 24, 3.60%), and others (n = 29, 4.35%) (Figure 1B). Among them, the main methods were electrical, visual, and direct brain stimulation.

#### Electrical stimulation

**Figure 3:**
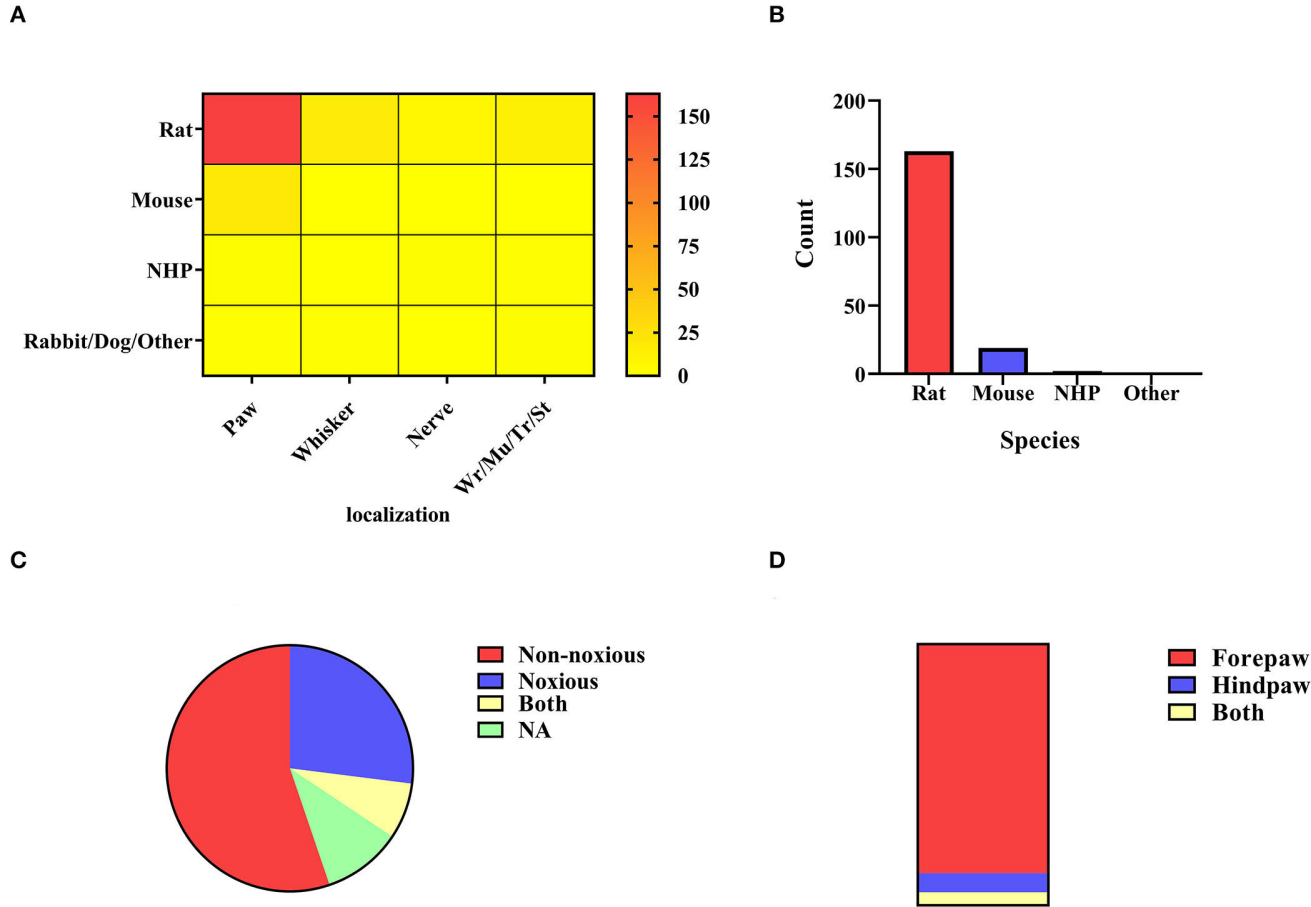
**(A)** Electrical stimulation localization. All electrical stimulation is classified according to the localization of stimulation, including paw stimulation (n = 183, 82.43%), whisker stimulation (N = 15, 6.76%), nerve stimulation (n = 9, 4.05%), and others (n = 15, 6.76%) including muscle, stomach, trunk, wrist. The color bar means the number of studies. **(B)** Paw stimulation. The species distribution of paw stimulation is rat (n = 161, 87.98%), mouse (n = 19, 10.38%), non-human primate (n = 2, 1.09%), and NA (n = 1, 0.55%). **(C)** Rats' paw stimulation. Noxious stimulation < 2 mA (n = 42, 26.09%) and non-noxious stimulation ≤ 2 m (n = 90, 55.90%). Some experiments covered both stimulus intensities (n = 12, 7.45%), and different stimulus intensities were adopted in the remaining chapters, or no specific description was given (n = 17, 10.56%). **(D)** Rats' paw localization. A bar graph was obtained by distinguishing the sites of stimulation by the forepaw (n = 141, 87.58%), the hind paw (n = 12, 7.45%), and both (n = 8, 4.97%).

The distribution of electrical stimulation sites and animal selection is illustrated in Figure 3A. It can be seen that rats (n = 194, 87.39%) were mostly used in the studies involving the electrical stimulation paradigm. As a simple and mature technology, electrical stimulation is widely used in the forepaw, hindpaw, and whiskers (Yu et al., 2016; Todd et al., 2019; Cywiak et al., 2020), in which the activated area is stable. The activated brain region for forepaw stimulation is the sensory cortex (somatosensory fore-limb region, S1FL) (Kim et al., 2005; Crofts et al., 2020), and the corresponding activated brain region for hind paw stimulation is the somatosensory hind-limb regions (S1HL) (Todd et al., 2019). Several studies have reported that whisker stimulation mainly activates the barrel cortex (S1BC) (Yu et al., 2012; Martin et al., 2013). It should be noted that there are various whisker stimulation methods, including mechanical stimulation and electrical stimulation. Whisker stimulation in this part refers to electrical stimulation of the whisker to achieve the activation effect. There were other stimulation methods, such as wrist electrode implantation to the median nerve, masticatory muscle implanted electrode to the trigeminal nerve (Just et al., 2010), stomach direct electrical stimulation (Yu et al., 2014; Cao et al., 2019), and trunk stimulation (Endo et al., 2008; Meuwissen et al., 2020). In short, electrical stimulation was set up in various ways with different blood oxygen fluctuations in the cortex. The cortex is the core region that responds to electrical stimulation (Sanganahalli et al., 2009).

##### Forepaw/hindpaw stimulation

In existing studies, electrical paw stimulation is a relatively mature stimulation paradigm, which has been applied to different animals (Figure 2A). Electrodes compatible with the MRI scanning system were implanted between the digits of the animals to apply stimulation, and the BOLD signals were observed in the brain regions or networks by setting different current parameters. The paw stimulation in rats was most widely studied (n = 161, 87.98%) (Figure 3B). The location of the rats' paw stimulation (Figure 3D) in most studies was the forepaw of rats (n = 141, 87.58%), and a small amount was in the hindpaw (n = 12, 7.45%). We further classified the paw electrical stimulation into noxious (n = 42, 26.09%) and non-noxious stimulation (n = 90, 55.90%) (Figure 3C). In the previous study, the threshold of the stimulating current was set at 2 mA. When the current was below the threshold value, only the contralateral corresponding sensory cortex was activated. Noxious forepaw stimulation greater than the threshold triggered activation of other brain regions associated with pain perception, including the secondary somatosensory cortex (S2), thalamus, insula, and limbic regions (Luo et al., 2009). Because of the species difference, the stimulating current threshold of mice (Luo et al., 2009; Adamczak et al., 2010; Jung et al., 2019) and non-human primates (NHP) (Luo et al., 2009; Qi et al., 2016; Yen et al., 2018) may not be the same. More detailed and targeted research studies are needed to determine the current threshold for different species.

##### Non-noxious and noxious electrical paw stimulation

As mentioned above, noxious electrical paw stimulation refers to a current intensity >2 mA. Due to its pain-causing characteristic, noxious electrical stimulation exceeding the stimulation threshold has been used in the study of a variety of inhibitors, like lidocaine. It has shown a decrease in global brain activation after lidocaine injection compared with the control group, suggesting that lidocaine may be a reasonable sedative and analgesic substance (Haile et al., 2019). A series of studies have investigated the CBF and BOLD signal changes in the bilateral striatum after the combined use of noxious electrical stimulation. The results show CBF reductions and reduced BOLD activation (Shih et al., 2011). Further studies are needed to explain the relationship between CBF and BOLD signal changes in the striatum and the current traumatic stimulation. For both noxious and non-noxious stimulation, the regions activated were the corresponding regions of the contralateral cortex areas, such as the contralateral primary somatosensory cortex. In a study on the cortex, S1 was divided into six layers, with the increase of current intensity (1–10 mA), cortical CBF in six layers also appeared in the change and was particularly pronounced in the II-V layer (non-noxious) and V-VI layer (noxious) of contralateral primary somatosensory cortex (Shih et al., 2013). The significance of noxious electrical stimulation is to create a visualized fMRI activation of pain-related regions (Pawela et al., 2017) and to study changes in cerebral blood flow (CBF) in the brain caused by pain inhibition or changes in pain intensity. Intuitive vascular and neural coupling observations are more convincing. Among paw stimulations, a class of studies investigated the effects of inhaled anesthetics on the intracerebral imaging of frequency-dependent paw stimulation, just like the effects of anesthesia on neural coupling mentioned above. Comparative experimental studies have also showed different BOLD signals under different anesthetics at the same frequency (Schroeter et al., 2014). Some studies have shown that anesthesia can also affect the adaptability of neural activity (Masamoto et al., 2007). This may indicate that anesthesia may modulate frequency-dependent sensory activation. The electrical stimulation at 3 Hz in chloralose anesthetized rats induced the greatest effect (Gyngell et al., 1996), while the maximum frequency of effect in isoflurane-induced rats was 8–12 Hz for a short period (10 s) and 6–8 Hz for an extended period (30 s) (Kim et al., 2010), which might provide a valuable reference for parameter setting of non-noxious paw stimulation.

Non-noxious electrical paw stimulation refers to a current intensity of < 2 mA, which generates stable activation in the brain regions. Compared with the wide-range activation of noxious stimulation, the activation range of non-noxious stimulation is limited, which was commonly used in the study of different disease models such as Alzheimer's disease model (Sanganahalli et al., 2013), stroke model (Sicard et al., 2006; Kim et al., 2007; Suzuki J. et al., 2013), and spinal cord injury model (Ramu et al., 2006, 2007). Neurovascular changes of model animals were reflected and compared through changes in blood flow in cortical areas by mapping brain activation after stimulation interventions. The non-noxious electrical stimulation had a stable paradigm. Therefore, it enabled comparisons of different anesthesia states (Verley et al., 2018), scan sequences (Seehafer et al., 2010), and contrast agents (Kim et al., 2005). Non-noxious electrical stimulation can be used as a more stable model to reflect changes in CBF and activation state induced by changes in external conditions. Interesting research about stimulating the bilateral ventricle and forepaw simultaneously showed differences in brain activation. This phenomenon may be related to the different afferent innervation of the heart, which overlaps with the clinical results of different ischemic lesions in the left and right ventricles (left or right ventricular myocardial ischemia) (Suzuki H. et al., 2013). By coupling nerve blood vessels, the research findings could provide evidence for the clinic.

##### Nerve electrical stimulation

Nerve stimulation was applied by inserting electrodes or placing electrical pads precisely into the target peripheral nerve regions (Figure 3A). The electrodes can be placed directly on the skin or surgically implanted. The main difference is that skin stimulation is pure sensory stimulation, while surgically implanted electrodes stimulate not only the sensory system but also motor activity and the deep brain structures (Cho et al., 2008). A few studies of direct nerve stimulation mainly involve stimulation of the trigeminal nerve (Cho et al., 2008; Just et al., 2010; Sonnay et al., 2017), the median nerve (Kennan et al., 1998, 2000; Hettinger et al., 2011; Liu et al., 2013). However, stimulation of multi nerves, such as the median, ulnar, radial, and musculocutaneous nerves, was occasionally reported. In this way, a distinct pattern of cortical activation was found with each nerve correlated with known sensorimotor afferent and efferent pathways to the rat forepaw (Cho et al., 2008). There were many related studies on median nerve electrical stimulation, some of which used the C7 nerve root transplanted rat model to evaluate the treatment effect through nerve stimulation to verify its clinical effectiveness (Stephenson et al., 2013). One study used implanted devices to study survival models in rats (Hettinger et al., 2011). The trigeminal nerve was stimulated by inserting the cathode into the suborbital hiatus and the anode into the masticatory muscle or neck muscle to observe the activation of S1BC in rats (Sonnay et al., 2017). Electrical stimulation was also performed on NHP, and greater activation of brain regions was observed in awake monkeys than in macaques under propofol anesthesia (Liu et al., 2013). The pathway of direct neural stimulation differs from sensory stimulation through the body surface to neural stimulation. Nerve stimulation is indeed a superior method to activate the brain regions of interest, which could avoid interference activation generated by other cortical sensory stimulation (Kida and Yamamoto, 2010).

##### Whisker pad electrical stimulation

As not all animal whiskers were susceptible to irritation, rodents were the primary subjects in the study on whisker electrical stimulation (Figure 3A). There were many ways of observing the changes in blood flow in the barrel cortex by stimulating the rats' whiskers, including pneumatic, mechanical, and electrical stimulation of the whisker pad. The whiskers were electrically stimulated on one side to alter the effect of the contralateral barrel cortex (Wehrl et al., 2014; Shih et al., 2021). In drug-related studies, Cheng et al. explored the effects of caffeine injection on the activation of the barrel cortex of whiskers in α-chloralose anesthetized rats (Shih et al., 2021). H2(15)O-PET and fMRI brain activation contrast experiments revealed that two kinds of brain activation maps can generate complementary physiological information, which is important for experimental research and clinical practice (Wehrl et al., 2014). These studies usually analyzed the neurovascular coupling (Hewson-Stoate et al., 2005; Devonshire et al., 2012), in which personal errors, such as the pressing of experimental animals' whiskers, should be avoided during scanning (Kida and Yamamoto, 2010).

##### Other electrical stimulation

The other types of electrical stimulation included wrist (Schwindt et al., 2004; Gsell et al., 2006; Hirano et al., 2018), trunk (Meuwissen et al., 2020), and stomach (Yu et al., 2014) (Figure 3A). For the wrist (Duricki et al., 2019) and trunk (Endo et al., 2008) electrical stimulation, electrodes were placed directly on the skin. For gastric stimulation, the abdomen is opened from the xiphoid process to expose the organs fully, and the electrode patch is implanted into the anterior stomach and then sutured. Gastric stimulation can drive somatosensory and cingulate cortices to generate extensive fMRI responses (Cao et al., 2019).

Although implanting electrodes in the stomach is difficult and risky, it is an effective way to stimulate nerves directly. Future studies could focus on the innervation effect of the stomach with electrical stimulation. The study on wrist electrical stimulation is not as valuable as that of median nerve stimulation implanted in the muscle, as the latter is more advanced in exploring the neurovascular coupling through fMRI f (see 3.3.1.3 Nerve Electrical Stimulation for more information) (Kida and Yamamoto, 2010).

Trunk stimulation has been carried out in studies on low back pain and spinal cord injury. The activation of brain regions associated with pain, such as the raphe nucleus, nucleus accumbens, and caudate putamen, can be observed by referring to the animal spinal cord stimulation model (T13) (Meuwissen et al., 2020). We can explore the mechanism of pain through different experimental designs and animal models, which may enhance our knowledge of pain and provide new insight into clinical treatment (Endo et al., 2008).

#### Visual and auditory stimulation

##### Visual stimulation

**Figure 4:**
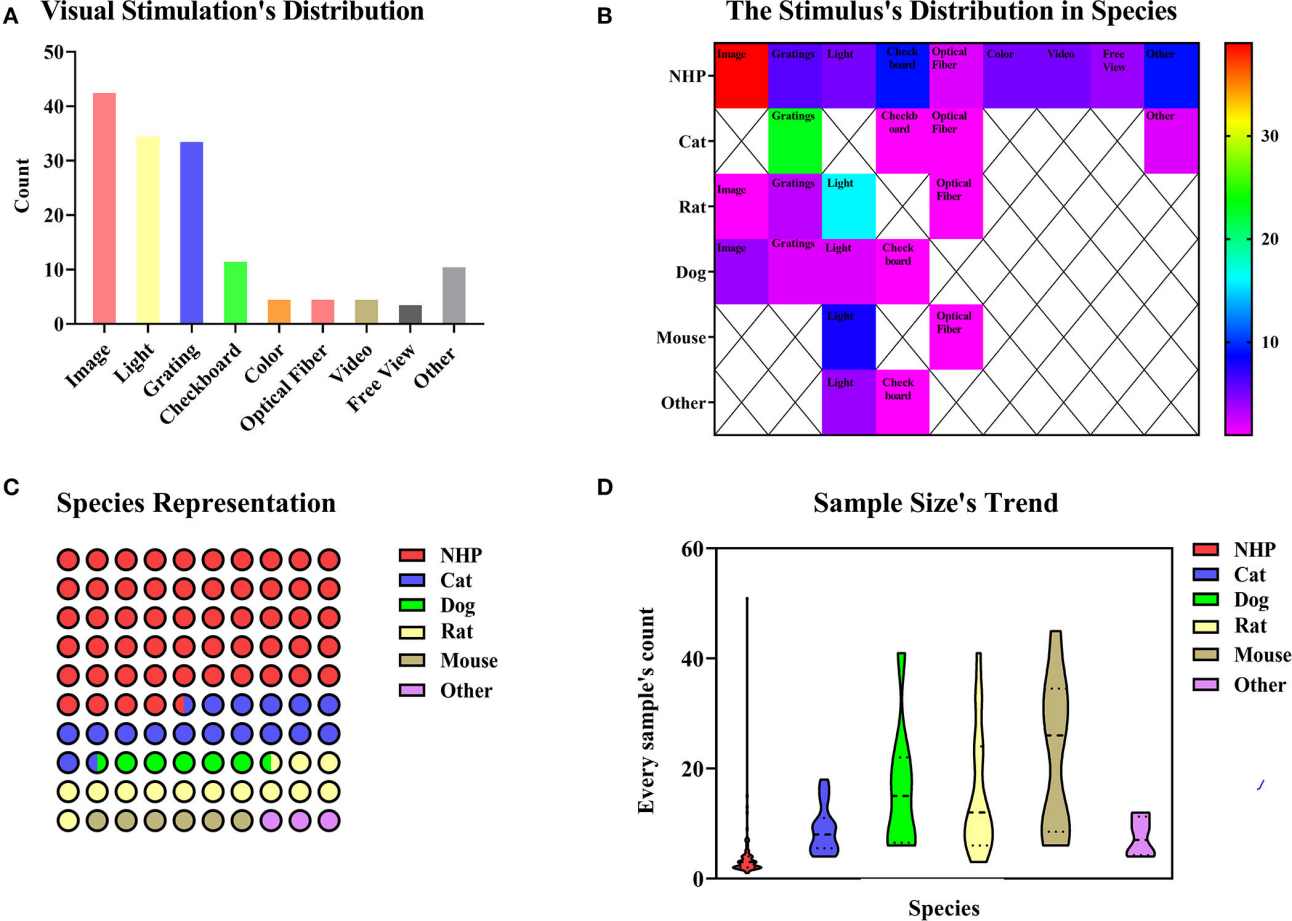
**(A)** In total of 154 paradigms of visual stimulation collected, we divided the methods of the stimulus into image (n = 43, 27.92%), light (n = 35, 22.72%), gratings (n = 34, 22.08%), checkboard (n = 12, 7.79%), color (n = 5, 3.25%), optical fiber (n = 5, 3.25%), video (n = 5, n = 5, 3.25%) and freeview (n = 4, 2.60%). Some experiments covered several different methods of the stimulus (n = 11, 7.14%). **(B)** Image is mainly used for NHP among the collected paradigms. Species distribution and sample size's trend. The color bar means the number of studies. **(C)** Animal representation in the collected paradigms. NHP, cats, and rats are the principal animals used for visual stimulation. **(D)** A number of animals used per fMRI study. NHP studies are carried out with fewer animals, whereas studies involving mice involve larger animals.

The visual stimulation paradigms use specific images, videos, light, or designed visual tasks to stimulate brain activities, which are observed during the fMRI scan. The stimulation methods can be classified according to different types of stimuli (Figure 4A). In 154 studies on visual stimulation paradigms, the types of stimuli included image (n = 43, 27.92%), light (n = 35, 22.72%), gratings (n = 34, 22.08%), checkboard (n = 12, 7.79%), color (n = 5, 3.25%), optical fiber (n = 5,3.25%), video (n = 5, 3.25%), and free view (n = 4, 2.60%). Some experiments combined several different visual stimuli (n = 11, 7.14%). Figure 4A shows images, light, and checkboards were the most commonly used visual stimuli. The stimulation's distribution in species highlights that the image is mainly used for NHP among the collected paradigms (Figure 4B). The BOLD signal changes induced by visual stimulation were mainly through gaze training of the awake or anesthetized NHP (Figure 4B), which combined the use of an anesthetic to relax the eye muscles and keep the eyes open (Dubowitz et al., 2001). The presentation of visual stimulation determined the different visual areas studied (Tsao et al., 2006; Lau et al., 2011a). Most studies used NHP as research animals, but there were also studies on rodent and cat models (Figure 4C). Meanwhile, we summarized the sample size distribution of visual stimulation experiments (Figure 4D). As with most animal experiments, the sample sizes for NHP experiments were generally < 10, while the sample sizes for other animal models varied. This sample size usually depended on the funding of the experiment and the difficulty of implementation. Since 1999, a repeatable and stable imaging method for visual stimulation based on BOLD signals has been explored (Logothetis et al., 1999). The implementation of visual stimulation requires pre-fixation training (Caspari et al., 2018; Premereur and Janssen, 2020) or keeping the animals' attention during the scanning process (Liang Z. et al., 2017; Xu et al., 2019) to achieve the desired stimulation effect. It is feasible to make the animals cooperate actively through training (Alizadeh et al., 2018). Another approach to forcing monkeys to stare at the target object was to keep the eyes passively open using a muscle relaxant (Jin and Kim, 2013). We also need to consider that different external visual signals present different stimuli that specifically activate different brain regions (Kaskan et al., 2017; Karl et al., 2020).

As an important signal of sensory input, vision is involved in many processes, such as facial recognition preferences in the brain (Russ and Leopold, 2015), the formation of reward mechanisms (Tsurugizawa et al., 2012), the processing of looming stimulation (Cléry et al., 2020), and 3D shapes. There are some classical brain regions activated (Tsurugizawa et al., 2010), but some other brain regions have been activated and reported in humans (Bunford et al., 2020), suggesting the value of studying the functional organization of the brain with visual stimulation.

##### Auditory stimulation

Auditory stimulation has been studied mainly in NHP and birds. Birds use calls to maintain social relationships, just as humans do with language (Van Ruijssevelt et al., 2018). Therefore, the audio types selected for auditory stimulation of birds were normally copulatory vocalization and songs of other birds (Voss et al., 2007; Maul et al., 2010). The auditory stimulation parameters in NHP were much more complex and varied. The stimulation paradigms may use human speech (Joly et al., 2012), infraspecific calls (Perrodin et al., 2011), extracted music (Wang et al., 2017), the “local-global” auditory paradigm (Uhrig et al., 2014), and broadband noise (Wong et al., 2017). These studies provide a critical basis for future studies of various functional properties of the animal auditory cortex.

As we discussed above, visual and auditory studies have used similar experimental paradigms to reveal the brain regions with specific functions. In the reported studies, many have compared the brain activations between humans and NHP to explore homology. The corresponding functional regions studied included the scene selection region (Nasr et al., 2011), the facial selection regions (Tsao et al., 2006), and the auditory cortex in a binocular blind rhesus monkey model (Wang et al., 2017). Visual or auditory stimulation can be applied as a single stimulation, but they are often combined in complex daily situations that involve the integration of higher-level complex information processing. Moreover, Guipponi et al. found that visual and auditory stimulation were closely combined and activated the superior colliculus (SC), which was of higher application value and more innovative in the study of higher-dimensional brain function (Guipponi et al., 2013; Lau et al., 2018). Research on single stimulation is also indispensable in scientific research. There were research studies on the auditory midbrain (Van der Kant et al., 2013), auditory forebrain (Maul et al., 2010), visual cortex (Boch et al., 2021), auditory cortex (Van Ruijssevelt et al., 2017), visual pathway (Leopold et al., 2002), new nerve nodes (Van Ruijssevelt et al., 2018), and nucleus functions (Lau et al., 2011b), which were the basis for further complex stimulation research.

#### Olfactory stimulation

**Figure 5:**
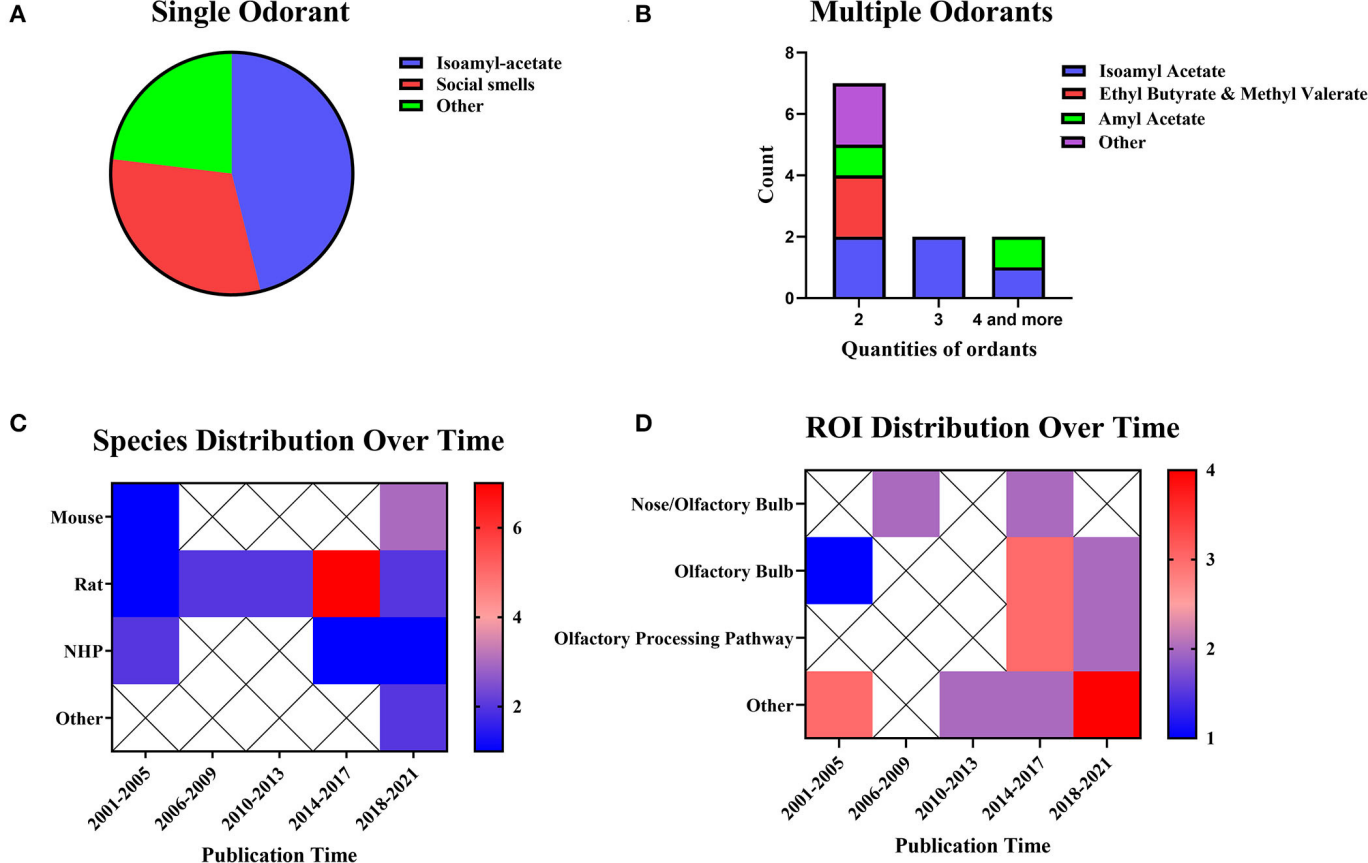
Odorants, species distribution, and ROI distributions. **(A)** Studies using single stimuli (n = 13, 54.17%). **(B)** Studies using multiple stimuli (n = 11, 45.83%). The color bar means the number of studies. **(C)** Temporal distribution of experimental animals. **(D)** Distribution of ROI over time. The color bar means the number of studies.

Olfactory stimulation refers to the delivery of a liquid or gas containing a specific odor to the area near an animal's nose through a certain transport route or carrier, thus activating the corresponding olfactory bulb region. The olfactory stimulation can be divided into single-odorant and multiple-odorant stimulation (Figures 5A,B). In both types of olfactory stimulation studies, isoamyl-acetate was the most commonly used stimulus. The rat was the most commonly used animal in olfactory stimulation experiments (n = 14, 58.33%), but the use of other animals has been increasing in recent years (Figure 5C). The olfactory bulb (n = 6, 25.00%) was the most popular ROI in olfactory stimulation experiments. Figure 5D shows a trend of multiple ROIs in recent years.

Olfactory stimulation cannot be implemented without an MRI-compatible taste transmitter. Animals are fixed on the scanning bed, and a transmitter sends specific odors within the animals' smell range. Specific odors include alcohol, almond flavor, amyl acetate (Poplawsky et al., 2015), ethyl butyrate, methyl valerate (Sanganahalli et al., 2016), various types of odoriferous chemicals (Zhao et al., 2018), and social smells of different species or the same species (Xu et al., 2005; Tikhonova et al., 2015) were sent through an MRI-compatible device. In olfactory stimulation experiments, it is also essential to design the parameters of odor transport location and transport time.

The current research has already shown evidence of the odor transport time and location. Some studies have observed the effects of olfactory stimulation of different time lengths (Martin et al., 2007). The analysis results showed the same effects of long-term and short-term stimulation. Similar research on NHP using 1/2/8 mins of stimulation revealed the same results. On the other hand, the research applied stimulation in different locations. Orthonasal and retronasal stimulation have shown overlapping response patterns and some route-specific dominance. Orthonasal maps were dominant in dorsal-medial regions, whereas retronasal maps were dominant in caudal and lateral regions (Sanganahalli et al., 2020). In summary, when applying olfactory stimulation, one should consider the odor type and the length of time. These results indicate that fMRI is the right choice for detecting the time and locations of olfactory bulb activation.

#### Direct brain stimulation

Direct brain stimulation means that the electrodes or fibers used for stimulation are implanted directly into specific areas of the brain regions to regulate the target pathways and brain regions directly. The main modes of action reported in relevant literature include DBS, EM, and optogenetics functional magnetic resonance (O-fMRI).

**Figure 6:**
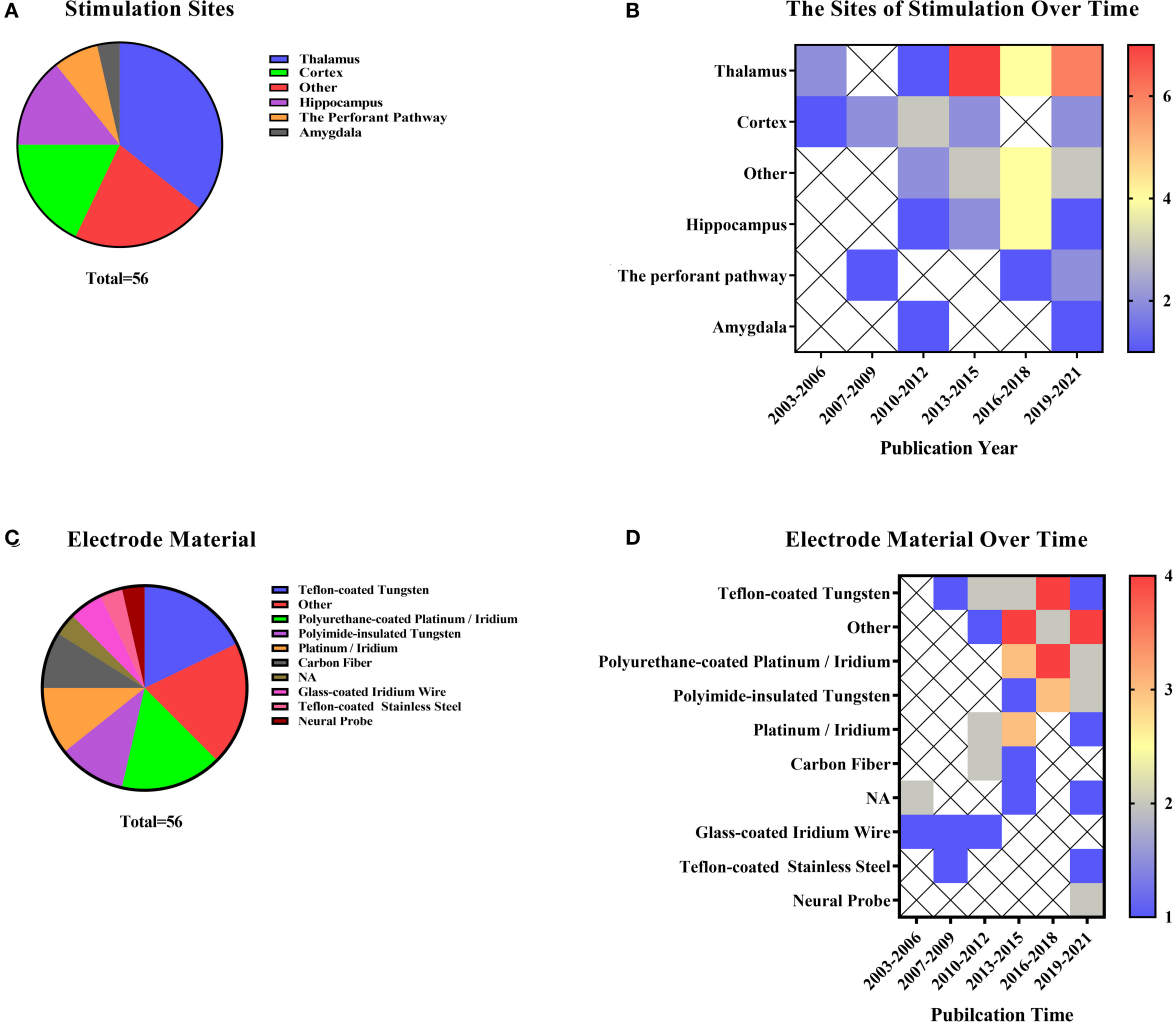
Direct brain stimulation includes deep brain stimulation, EM, and optogenetics **(A)** Stimulation site representation distribution and electrode material distribution in deep brain stimulation and micro-current stimulation. **(B)** Distribution of stimulation sites over the last two decades. The color bar means the number of studies. **(C)** Distribution of stimulating electrode materials used in articles related to direct brain stimulation. **(D)** Electrode materials occur in the literature over time. The color bar means the number of studies.

Previous studies have not clearly distinguished between brain EM and DBS (Jiang et al., 2015). During fMRI scans, implanted objects may interfere with data collection (Shyu et al., 2004). The different locations of electrode implantation are summarized in Figure 6A, and its year-to-year distribution is described in Figure 6B. Meanwhile, researchers need to consider selecting MRI-compatible electrodes before applying stimulation. In the DBS and EM animal studies, the electrode materials selected are shown in Figure 6C. Teflon-coated tungsten (n = 10, 17.86%) was the most commonly used electrode material. Other materials included graphene fiber, non-magnetic gold electrodes, iridium, and more. In recent years, more and more new materials have been used to reduce artifacts (Figure 6D). The thalamus (n = 20, 35.71%) was a popular site for deep brain stimulation and micro-current stimulation experiments. Other stimulating areas included the deep cerebellar nuclei, lateral olfactory tract, and more. Besides, some studies may involve two stimulating areas. Hippocampus (n = 8, 14.29%) has been a popular stimulating region in the last decade in this research field.

**Figure 7:**
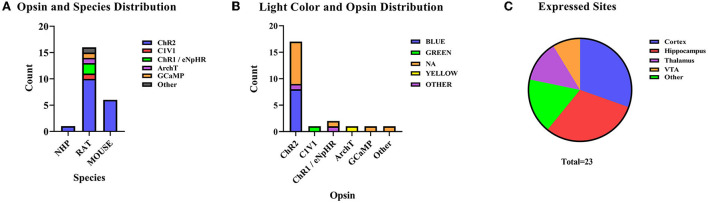
As for optogenetics fMRI (O-fMRI). **(A)** Distribution of animal species in O-fMRI, ChR2 is the most commonly used optogenetics protein. **(B)** The distribution of color of stimulating light and opsin in documented studies. **(C)** The distribution of sites of opsin expression.

O-fMRI refers to the use of functional magnetic resonance to reflect certain activated or suppressed pathways or circuits in optogenetics-regulated animals. Usually, the experimental procedures for optogenetics include stereotactic injection, fiber implantation, and post-implantation stimulation, with specific photosensitive viruses implanted at corresponding intracerebral coordinates (Just and Faber, 2019). Optogenetic techniques can be a possible way to stimulate the brain regions through light-sensitive channels (Kahn et al., 2011). Different laser emitters activated the light-sensitive channels, mainly a blue laser at 473 nm and some blue and yellow (593 nm) pulsed lasers as well (Takata et al., 2015). Channel protein distribution and laser activation are described in Figures 7A,B. Rats (n = 16, 69.57%) were the most commonly used animals in O-fMRI. The opsin injection site was also collected (Figure 7C). The cerebral cortex (n = 7, 30.43%) and hippocampus (n = 7, 30.43%) have been hot spots for research. Studies mainly explore various brain regions in the cortex, such as the medial prefrontal cortex, neocortex, and S1FL.

EM and DBS are highly similar. EM uses fMRI to observe the neurovascular changes in specific brain areas through electrical stimulation alone or in combination with other stimulations. When combined with certain behavioristics, it can be verified in reality and in the brain (Murris et al., 2021). In the studies on EM and DBS in animals, the selection of different electrodes and stimulation sites deserves our attention. The current intensities of EM and DBS were low and directly affected the brain regions. Weak current (microampere) stimulation was generated for the brain regions and pathway, and changes in blood oxygen levels in brain regions corresponding to this pathway can be observed. Yang compared the functional connectivity of the lateral and medial thalamic cortical pain pathways in normal rats by using a BOLD activation pattern induced by direct electrical stimulation of the posterior ventral (VP) and middle temporal region (MT) regions of the forebrain. Such stimulation experiments directly analyzed whether there were fundamental differences in signal processing between the medial and lateral thalamic pathways (Yang et al., 2013).

Due to the selectivity of regulating channel proteins and the numerous circuits and regions of interest in the brain, the BOLD changes in the brain can be visually observed in combination with optogenetics, thus achieving the goal of observing neurovascular coupling. Regarding the selection of channel proteins, different researchers used the same opsin to activate the ROI, such as using CHR2 to activate dHP and vHP (Wang et al., 2019); S1FL (Schmid et al., 2016, 2017; Uhlirova et al., 2016); M1 AND CPU (Ryali et al., 2016). Other studies selected inhibitory channel proteins such as eNpHR and ARCHT to inhibit frontal eye field neurons (Ohayon et al., 2013). eNpHR can be verified in both activation and inhibition (Liu et al., 2015). In conclusion, the diversity of photogenetic techniques combined with functional magnetic resonance can effectively reflect the accuracy of brain pathways or connections from the neurovascular coupling.

#### Mechanical stimulation

**Figure 8:**
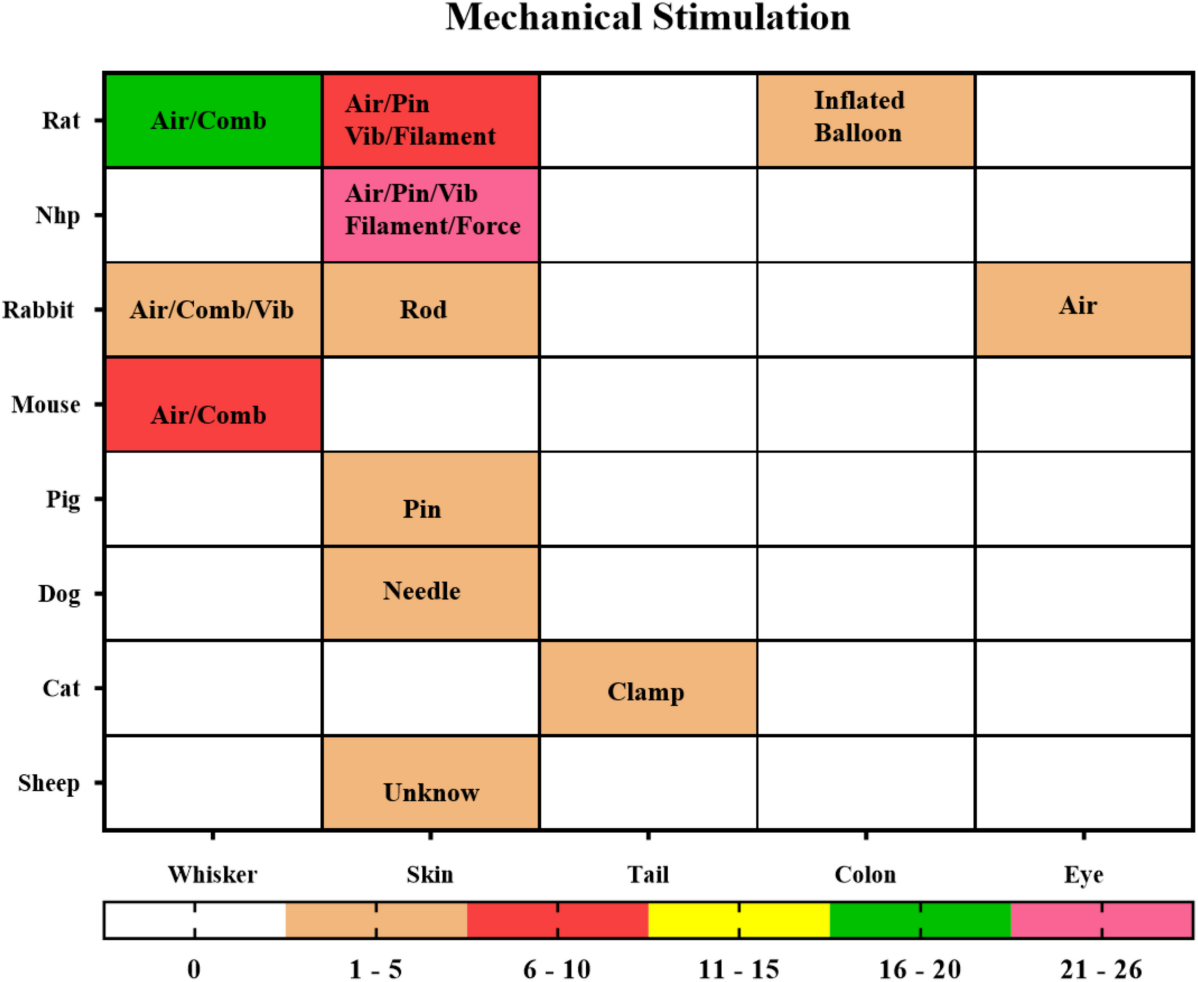
Mechanical Stimulation. The locations of stimuli and species distribution are displayed through a hotspot map, and the content in the hotspot grid is the medium used to apply the stimuli, including air, pin, vibrator (Vib), filament, force, clamp, comb, balloon. The color bar means the number of studies.

Mechanical stimulation refers to the compression of the skin of an animal by air, a drive device, comb, pin, rod, or clamp to produce a relatively simple effect of pure mechanical stimulation, including the classic monofilament skin stimulation, pneumatic skin stimulation, whisker stimulation, and vibrator sticking to the skin. The conduction pathways of skin stimulation are relatively simple, as are their stimulation methods and species distribution (Figure 8). The most common stimulation methods are applying pneumatic equipment, plastic spin, filament, vibrator, and force on the skin of NHP, rodents, and other animals (n = 39, 54.17%). Studies on whisker stimulation were also common (n = 27, 37.5%), and the species were limited. Further studies included intestinal pressure stimulation, eye gas stimulation (Miller et al., 2003), and tail clamping stimulation (Nagakubo et al., 2017), which can be performed in a variety of easy and repeatable ways.

The intelligence of mechanical stimulation is in the application and design of the stimulation, such as the use of a balloon implanted in the colon to inflate the intestine of the rat to achieve the stimulation effect of intestinal obstruction with gas (Lazovic et al., 2005; Tsurugizawa et al., 2009a). This is an effective way to mimic colorectal distention.

Abdominal pressure stimulation was used to simulate menstrual pain in NHP (Yano et al., 2019); a vibrator was used on the NHP forearm to create tactile stimulation for the fingers (Zhang et al., 2007, 2010); blowing into the eyes of rabbits was to induce blinking (Miller et al., 2008); clamping the tail of cats with nylon wire was to produce painful mechanical stimulation (Nagakubo et al., 2017); squeezing the right hind leg muscle was used to trigger BOLD change. All these stimulation methods can be used as reasonable and repeatable paradigms in experiments.

In addition to the electrical whisker stimulation mentioned in the part on electrical stimulation, there were other ways to stimulate the whisker barrel cortex in rodents through mechanical stimulation, such as using a mechanically driven comb or pneumatic connector (Lu et al., 2003, 2004, 2016). Mechanical stimulation could be achieved in a variety of ways, but the conduction pathway is relatively fixed. Different stimulation methods are suitable for different ROIs, so we should consider study aims and nuclear magnetic compatibility to select the appropriate stimulation methods in the study design.

#### Multiple stimulations

Single stimulation research started early. After a certain research foundation was established, the scholars turned to another blank area to explore the fMRI brain activation mode under the combination of various stimulation paradigms. Various combined stimulation studies have been carried out in the past decades, which can be seen from the distribution of time points (Figure 1A). The combination of stimulation is sometimes not merely a superimposed benefit but rather a multichannel study (Lambers et al., 2020) of peripheral stimulation delivery equipment required to produce the same stimulation inside and outside the magnet to simulate a more realistic environment. Studies of multisensory convergence of dynamic cortical activation with information integration processing and complex sensory processing regions depend on input from various stimulation modes (Sanganahalli et al., 2009). Similarly, the repeatability of studies also depends on constant stimulation and specific activation of brain regions, which is true in single-stimulation studies and should be true in multistimulation studies. In addition, the study on the multistimulation mode is more consistent with the process of information processing and environmental interaction in daily life (Cook et al., 2016; Cléry et al., 2017; Arsenault and Vanduffel, 2019), resulting in a coherent perception of the environment (Kayser et al., 2005). With optogenetics techniques and EM, some stimulation tasks that are difficult to complete in MRI scans can be modulated by these brain stimulation techniques to achieve the same benefits (Poplawsky et al., 2015; Leong et al., 2019). As mentioned above, the bidirectional regulation of optogenetics techniques has been combined with other stimulations. The comparison of the regulatory results of optogenetics combined interventions and EM intervention alone (Schmid et al., 2017) is more convincing (Ekstrom et al., 2008, 2009). With the combination of technologies and innovations, the research technology of magnetic resonance has become more extensive, which may also be a direction for future research.

#### Other stimulation

In the study on the stimulation paradigm, in addition to the above studies that can be clustered, some sporadic niche stimulation is classified as other stimulation, which includes chemical, thermal, and food stimulation. The chemical stimulation should consider the selection of chemical substances and the injection site. For example, some experiments injected cocaine during the scan (Mandeville et al., 2001, 2004), or the pain-causing substances into the forepaw or directly created a hyper-carbonated environment to cause hypoxia in the brain (Kannurpatti et al., 2003). In addition to the internal chemical stimulation, stimulation can also be applied to external sites. Pepper spray applied directly to the paws can have the same effect as electrical or mechanical stimulation (Kannurpatti et al., 2002).

Intragastric administration can direct food stimulation to observe BOLD changes in the brain regions associated with intestinal stimulation, such as the amygdala, hippocampus, and the ventral tegmental area (Tsurugizawa et al., 2009a,b, 2010). There are differences among various stimulation paradigms, but there are many overlaps in BOLD changes caused by stimulation and the selection of ROI (Mazzanti et al., 2011). Researchers can apply appropriate stimulation methods to the animal models to achieve their research goals.

## Conclusion

The collection and analysis of animal functional magnetic resonance combined with stimulation show that anesthesia imaging still occupies the mainstream, and awake imaging studies have gradually appeared in recent years. Besides, the stimulation methods have gradually broadened with the progress of science and technology. Various devices and scanning technologies with high resolution and high specificity are constantly emerging, which makes the research more detailed. It is critical to choose a suitable paradigm combined with the research objectives, including the selection of stimulus mode and the setting of various parameters. Besides, a more innovative paradigm needs to be created and explored for animal functional magnetic resonance combined with stimulation needs to meet the demands of investigating the brain in future studies.

## Data availability statement

The raw data supporting the conclusions of this study are available on request to the corresponding authors.

## Author contributions

SL, JH, YZ, and QZ contributed to conception and design of the study. JH, YZ, QZ, LW, XZ, CJ, JY, and ZL collected the data. JH, YZ, and QZ wrote the first draft of the manuscript. SL revised the manuscript. All authors contributed to manuscript revision, read, and approved the submitted version.

## Funding

This study was supported by the grants from the National Natural Science Foundation of China (82004440 and 81803883), the Natural Science Foundation of Fujian Province (2021J01961), the Youth Science Foundation of Fujian Provincial Health Commission (2019-1-65), and the Scientific Research Foundation for the High-level Talents funded by Fujian University of Traditional Chinese Medicine (X2019002-talents).

## Conflict of interest

The authors declare that the research was conducted in the absence of any commercial or financial relationships that could be construed as a potential conflict of interest.

## Publisher's note

All claims expressed in this article are solely those of the authors and do not necessarily represent those of their affiliated organizations, or those of the publisher, the editors and the reviewers. Any product that may be evaluated in this article, or claim that may be made by its manufacturer, is not guaranteed or endorsed by the publisher.
